# Trust in automated vehicles: constructs, psychological processes, and assessment

**DOI:** 10.3389/fpsyg.2023.1279271

**Published:** 2023-11-23

**Authors:** Francesco Walker, Yannick Forster, Sebastian Hergeth, Johannes Kraus, William Payre, Philipp Wintersberger, Marieke Martens

**Affiliations:** ^1^Cognitive Psychology, Leiden University, Leiden, Netherlands; ^2^BMW Group, Munich, Germany; ^3^Johannes Gutenberg University Mainz, Mainz, Germany; ^4^Coventry University, Coventry, United Kingdom; ^5^TU Wien, Vienna, Austria; ^6^University of Applied Sciences Upper Austria, Hagenberg, Austria; ^7^Industrial Design, Eindhoven University of Technology, Eindhoven, Netherlands

**Keywords:** trust, trust in automation, automated driving, self-driving, trust calibration, human factors, automated vehicles, SAE levels

## Abstract

There is a growing body of research on trust in driving automation systems. In this paper, we seek to clarify the way trust is conceptualized, calibrated and measured taking into account issues related to specific levels of driving automation. We find that: (1) experience plays a vital role in trust calibration; (2) experience should be measured not just in terms of distance traveled, but in terms of the range of situations encountered; (3) system malfunctions and recovery from such malfunctions is a fundamental part of this experience. We summarize our findings in a framework describing the dynamics of trust calibration. We observe that methods used to quantify trust often lack objectivity, reliability, and validity, and propose a set of recommendations for researchers seeking to select suitable trust measures for their studies. In conclusion, we argue that the safe deployment of current and future automated vehicles depends on drivers developing appropriate levels of trust. Given the potentially severe consequences of miscalibrated trust, it is essential that drivers incorporate the possibility of new and unexpected driving situations in their mental models of system capabilities. It is vitally important that we develop methods that contribute to this goal.

## 1 Introduction

Automated vehicles are expected to drastically reduce road accidents, minimize the workload associated with driving and increase traveling comfort, allowing drivers to engage in various activities (so-called “non-driving related tasks,” or NDRTs) while the car takes care of driving (Fagnant and Kockelman, 2015; Payre et al., 2016; Kyriakidis et al., 2017; Milakis et al., 2017; Van Nes and Duivernvoorden, 2017; Litman and Litman, 2023). At the same time, the introduction of more advanced automated driving technology creates new challenges for human-machine interaction—many of which can be traced back to the way users interact with the technology (Lee and See, 2004; Kyriakidis et al., 2017; Carsten and Martens, 2019; OVV, 2019; Wintersberger et al., 2021; NHTSA, 2022; Zhang et al., 2022).

Various parties have raised concerns that driver assistance systems and Automated Driving Systems (ADS) may have unintended side effects, such as inappropriate driver trust and over-reliance on automation (Mueller et al., 2022). Organizations like NHTSA (2022) and the Dutch Safety Board (OVV, 2019) have reported and analyzed crashes and fatal accidents directly related to the use of—and overtrust in—this technology.

In theory, a system that never fails should always be trusted. However, achieving 100% reliability in complex automated systems is unfeasible. Additionally, trust, as a psychological construct, is significantly influenced by subjective factors, which are not always in line with objective reliability (Lee and See, 2004; Hergeth et al., 2016). As early as 1983, Bainbridge identified the “ironies of automation,” which include over-reliance on automated systems, skill loss, and reduced vigilance. Studies investigating these ironies of automation have clearly demonstrated that “the more advanced a control system is, the more crucial the contribution of the human operator” (Bainbridge, 1983, p. 775). In other words, the higher the reliability of an automated system, the more substantial the Human Factors challenges become (Bainbridge, 1983; Parasuraman and Riley, 1997; Kyriakidis et al., 2017; Boelhouwer et al., 2019; Carsten and Martens, 2019; Frison et al., 2019; Walker, 2021).

Previous experience with automated systems suggests that their effectiveness depends not only on the technology itself but also on the level of trust humans place in them (Lee and See, 2004; Hoff and Bashir, 2015; Schaefer et al., 2016; Kyriakidis et al., 2017). This holds equally true for automated vehicles. Indeed, many driving situations exist where suboptimal human-machine interaction can lead to potentially hazardous outcomes (Lee and See, 2004; Saffarian et al., 2012; Martens and van den Beukel, 2013; Kyriakidis et al., 2017; Carsten and Martens, 2019; Nees and Liu, 2022).

Trust in automated vehicles depends on a broad range of factors, including vehicle behavior, workload and the predispositions of the driver toward the automated system. Furthermore, several authors have suggested that diminished situational awareness, combined with increased in drivers' response times and unexpected vehicle behavior, will have a strong impact on the safety of automated driving technology (Sarter et al., 1997; Saffarian et al., 2012; Martens and van den Beukel, 2013; De Winter et al., 2014; Carsten and Martens, 2019).

More generally, as automated driving technology becomes more reliable and Operational Design Domains (ODDs) are extended, driver misconceptions concerning the automated vehicle's capabilities are likely to increase. This could potentially lead to underestimation of the probability and consequences of an automation failure (Seppelt and Victor, 2016; Flemisch et al., 2017; Victor et al., 2018; Wagner et al., 2018; Carsten and Martens, 2019; Holländer et al., 2019).

Loss of situational awareness and slow or inadequate human response in case of automation failures can often be interpreted as an excess of trust, or “overtrust” (also described as “complacency”; Muir, 1987; Parasuraman et al., 1993; Parasuraman and Riley, 1997; Lee and See, 2004; Inagaki and Itoh, 2013; Hoff and Bashir, 2015; Payre et al., 2016; Boubin et al., 2017; Flemisch et al., 2017; Noah et al., 2017; Lee et al., 2021; Lee and Ji, 2023). However, there are also situations in which users do not place enough trust in a reliable system (Muir, 1987; Parasuraman and Riley, 1997; Lee and See, 2004; Hoff and Bashir, 2015; Carsten and Martens, 2019). Some authors have called this “undertrust” (or “distrust”) (Muir, 1987; Lee and See, 2004; Sheridan et al., 2005; Hoff and Bashir, 2015; Wintersberger et al., 2018; Lee and Ji, 2023).

Against this background, this paper departs from key constructs used in analyses of trust in automated vehicles. We then proceed to discuss the underlying theories and the key psychological processes involved in the formation, calibration and measurement of trust. While the views of the authors sometimes diverge on specific details, we agree that trust plays a pivotal role in the safe deployment of current (Level 2, Level 3) and future (Levels 4 and 5) commercially available Automated Driving Systems (ADS) and that the literature often presents an over-simplified view of what this means. In this respect, users are often depicted as either trusting or not trusting a system; unfortunately, we still observe instances where researchers aim for maximum trust, regardless of the technology, overlooking the crucial aspect of trust calibration. Against this background, our goal in this overview paper is to provide a comprehensive and nuanced discussion of theoretical and methodological considerations as a foundation for theoretically sound research on users trust and their interaction with ADS.

More specifically, we will discuss:

- The role of drivers' trust in automated driving, and how this changes depending on ADS.
- The complex and multi-layered character of trust.
- The importance of trust calibration.
- Best practices and procedures currently used to measure trust.
- Key challenges for future research on trust in the domain of automated driving.

## 2 Trust in automated vehicles

Researchers interested in the relationship between individuals and automated agents use concepts borrowed from studies of interpersonal (human to human) trust (e.g., Walker et al., 2018; Kraus, 2020). The most widely adopted definition comes from Lee and See (2004), who define trust as “the attitude that an agent will help achieve an individual's goals in a situation characterized by uncertainty and vulnerability” (p. 51). Here, we highlight the key aspects of trust in automated vehicles:

*Trust in automated driving functions refers to road users'* ***subjective*** *evaluation of the ability of an automated vehicle to drive safely*. ***Trust is a multi-layered concept, combining different*** ***trust variables, namely dispositional trust, initial learned trust, situational trust*** ***and dynamic learned trust*. ***Trust is the result of a* ***dynamic psychological process*** *that varies over time, depending on the driving scenarios and users'* ***experience***.

While we cannot directly observe the level of trust, we can observe its behavioral outcomes, such as reliance. Trust is neither a unidimensional (someone either trusts or does not trust) nor a categorical construct. Reluctance to use reliable automation (distrust or undertrust) and its misuse (mistrust or overtrust) can be seen as two extremes along a continuum. Drivers' position on this continuum may fluctuate due to various factors, including knowledge, expectations, and the perceived reliability of the vehicle. Therefore, trust is **dynamic**: it develops and changes over time depending on driver experience, their individual learning history with the system, and the specific conditions at hand (Kraus et al., 2019; Kraus, 2020; Walker, 2021).

**Experience** cannot be measured exclusively in terms of time or kilometers driven. It should also take account of the range of situations experienced by the driver. Thus, drivers who have only experienced the automated system in a small set of driving scenarios (e.g., when a car stays in-lane as it traverses a wide curve on a motorway), may still be considered inexperienced, even if they have repeatedly encountered such situations. This is because their mental model, and therefore their cognitive representation of vehicle capabilities (Rouse and Morris, 1986; Nees and Liu, 2022), is based on a limited range of experienced driving scenarios. The more diverse and fine-grained the driving situations a driver encounters, the richer their experience becomes. Consequently, the driver's mental model becomes more valid (e.g., the driver may learn that the car will stay in-lane only if certain conditions are met). Overall, we argue that “experience” should not be measured in units such as time spent with a system or a particular distance traveled, but in the number of mutually exclusive situations in which an operator can assess the behavior of the system.

Ultimately, researchers and developers should not strive for the highest level of trust, but rather for calibrated trust (Walker et al., 2018; Wintersberger et al., 2020). This means that, ideally, drivers' position on the disuse-misuse continuum should be continuously aligned with the actual reliability of the automated system in the current situation.

### 2.1 Trust development

In the literature, the development of trust in an automated system is often described as a learning process, involving a small number of key constructs. For example, Marsh and Dibben (2003), followed by Hoff and Bashir (2015), proposed three interdependent trust layers: dispositional trust, situational trust and learned trust.

*Dispositional trust* reflects the operator's tendency to trust automation in general (Hoff and Bashir, 2015; Kraus et al., 2021). As a stable trait existing *prior* to interaction with the automated system, it is influenced by factors such as age (e.g., Schoettle and Sivak, 2014a; Abraham et al., 2016; AAA, 2018), gender (e.g., Payre et al., 2014; Hulse et al., 2018), culture (e.g., Schoettle and Sivak, 2014a,b; Hergeth et al., 2015) and personality (e.g., Payre et al., 2014; Choi and Ji, 2015; Kraus et al., 2021).

*Situational trust* refers to the trust shown by a user of an automated system, in a specific situation at one specific time. It depends on the user's context-dependent characteristics (e.g., self-confidence), on the specifics of the situation (e.g., overtaking), the general characteristics of the environment (e.g., weather, light, and road conditions) and the behavior of the system in that situation (Lee and See, 2004; Rovira et al., 2007; Hoff and Bashir, 2015; Hergeth et al., 2016; Carsten and Martens, 2019; Holthausen et al., 2020). Notably, situational trust develops in relation to specific events. The set of such events affects the user's (dynamic) learned trust. Therefore, although (dynamic) learned trust and situational trust are both influenced by experience, the former develops through the latter (Marsh and Dibben, 2003).

Finally, *dynamic learned trust* is the trust that users develop during system use, based on the skills and knowledge acquired through past experiences and interactions with the system. In contrast to situational trust, which is per definition strongly situation-specific, dynamic learned trust is more general and thus is established and calibrated gradually as the user acquires more knowledge about the system's capabilities and performance (Hoff and Bashir, 2015; Kraus, 2020; Walker, 2021).

Although Hoff and Bashir's (2015) model did not specifically focus on automated driving technology, numerous studies investigating trust toward automated vehicles refer to their work (e.g., Hergeth et al., 2016; Haeuslschmid et al., 2017; Habibovic et al., 2018; Körber et al., 2018; Kraus et al., 2019; Holthausen et al., 2020; Lee and Kolodge, 2020). In the same spirit, several of the authors of this manuscript (see Kraus, 2020; Walker, 2021) have used modified versions of Hoff and Bashir's (2015) trust model to explain how trust toward automated (driving) technology develops over time.

While valuable, we believe that Hoff and Bashir's (2015) model could be revised. In particular, we find the lack of a clear distinction between situational and learned trust to be problematic. When researchers interpret these terms differently or use them interchangeably, the result is confusion in the literature. In what follows, we question how far Hoff and Bashir's (2015) terminology adequately describes the evolution of trust in automated driving (but also other) systems over time.

**Figure 1:**
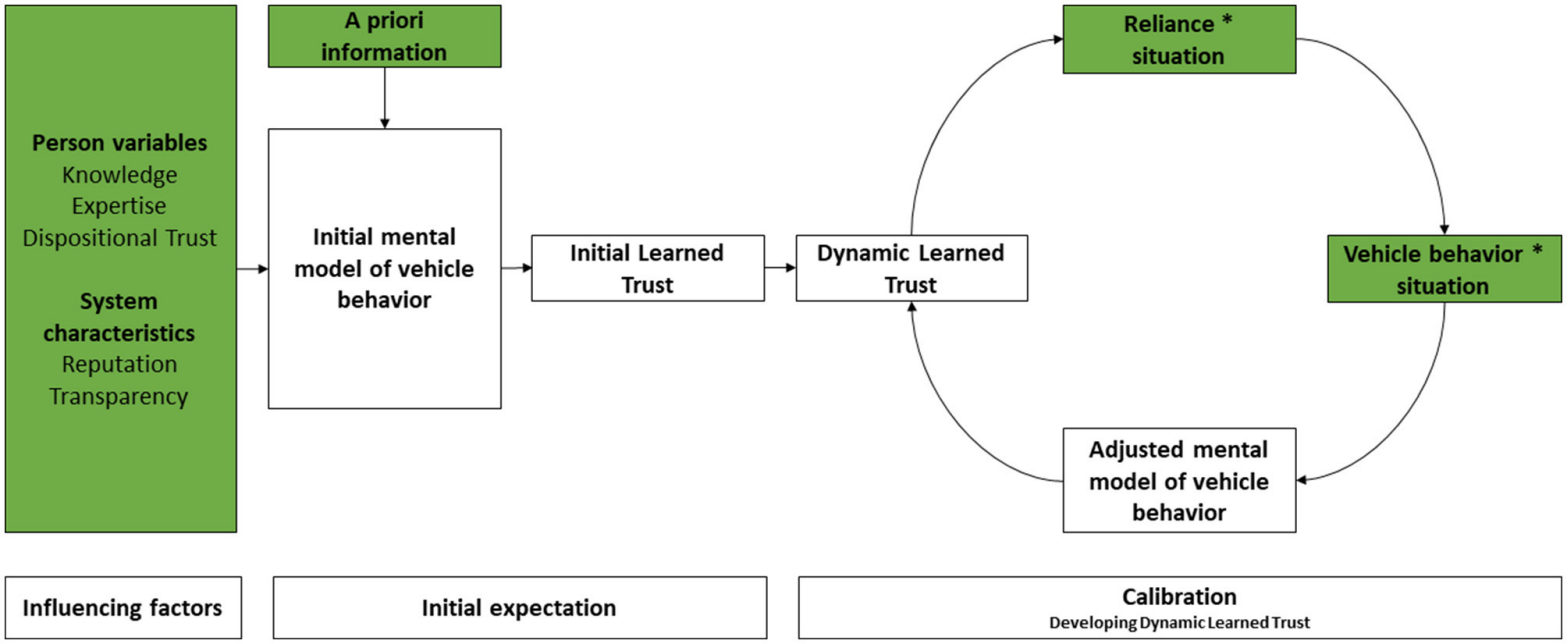
A conceptual framework of the development of trust toward a specific automated driving system (based on Lee and See, 2004; Ghazizadeh et al., 2012; Hoff and Bashir, 2015; Kraus, 2020; Walker, 2021). * = By.

To clarify this perspective, we distinguish between expectations (which encompass dispositional and initial learned trust) and calibration (which includes situational and dynamically learned trust, i.e., all experiences derived from interacting with a specific system in various situations). As a result, we propose a simpler framework with revised terminology and provide a use-case example demonstrating its application. In this framework (see Figure 1), observable factors and actions are highlighted in green.

Naïve drivers develop an *initial mental model of vehicle* behavior. Their initial model is influenced by several factors, including dispositional traits (e.g., personality) and what has been learned from the media and people with whom drivers have contact (e.g., Kraus, 2020). This model shapes the driver's expectations, thereby establishing a foundation for trust (i.e., *Initial Learned Trust*) before any interaction with the automated driving system.

When using the system for the first time, drivers Initial Learned Trust will correspond to their *Dynamic Learned Trust*. The latter affects the decision of the driver to *rely* on the features of the automated vehicle to a greater/lesser extent in specific situations. In turn, the driver's *reliance*—an observable action—will impact the behavior of the automated driving system.

Finally, after *observing the vehicle's behavior in a specific situation*, drivers evaluate whether it was appropriate or not to rely on the automated system, and *adjust/calibrate* (see section 2.2) their mental model of vehicle behavior accordingly (Forster et al., 2018; Kraus, 2020; Wintersberger et al., 2020; Walker, 2021). To illustrate the relation between these variables, please consider the following use-case scenario (adapted from Beggiato et al., 2015).

In an on-road study, 15 drivers were asked to use Adaptive Cruise Control (ACC) for the first time. ACC is an advanced driving assistance system (ADAS) that partly automates longitudinal car control while maintaining a constant safety distance from a leading vehicle. Importantly, sensor limitations can hinder the use of ACC, and therefore drivers may be required to manually take back control of the car (Beggiato et al., 2015).

Within a 2-month period and for 10 times, all participants drove a vehicle equipped with ACC on a predefined route. Although drivers had not experienced ACC before (*influencing factors*), they were asked to read the ACC-section of the owner's manual (*a priori information*). Through this information, drivers developed a *mental model of vehicle behavior*, and therefore initial expectations toward the system (*Initial Learned Trust*).

Beggiato et al.'s (2015) results showed that *Dynamic Learned Trust*, measured via the Trust in Automation scale (Jian et al., 2000), grew steeply after the first session and reached a stable level after the fifth session. Throughout the study, no substantial trust declines were observed. Overall, drivers relied (*reliance* ^*^ *situation*) on ACC (*vehicle behavior* ^*^ *situation*) in multiple situations (e.g., different bends, speeds and weather conditions), and *adjusted their mental model* accordingly.

The development of an appropriate level of trust can be viewed as a feedback cycle in which the driver—by interacting with the system in multiple situations—learns how the vehicle behaves and, therefore, when it can be trusted and relied upon (i.e., used; Kraus, 2020; Walker, 2021). Importantly, although potential limitations of the system that have not been encountered on the road will be dropped from the user's mental model (Beggiato et al., 2015), this feedback cycle can lead to more reliable inferences concerning both experienced and unexperienced scenarios (Walker et al., 2018). We will continue discussing this dynamic process in the following section.

### 2.2 Trust calibration

Trust calibration is a dynamic mental process shaped by experience and beliefs, allowing an individual's trust levels to vary depending on automation capabilities (Muir, 1987; Lee and Moray, 1994; Lee and See, 2004). In practice, trust calibration is the *assessment* of the balance between trust and the automation capabilities. In theory, it is the *objective measurement* of that balance. We argue that *assessing* the balance between trust and the automation capabilities offers more insights into how, why and when individuals use automation, compared to its *objective measurement*. The following examples depict how trust calibration is more often evaluated than objectively quantified within the context of automated driving research. Trust can be well calibrated regardless of automation performance. For instance, drivers preparing to resume the Dynamic Driving Task (DDT) performance when they expect the ADS is about to issue a request to intervene might be considered a good calibration of trust. Conversely, distrusting an automated vehicle that drives safely and complies with the road legislation indicates poor calibration, or miscalibration.

These examples illustrate that calibration of trust is achieved via an assessment rather than the application of objective and quantifiable measures. Calibration is optimal when a user's level of trust matches the capabilities of the automation. Optimal and accurate calibration of a user's trust relative to an automated system's capability occurs over time when interacting with and experiencing boundary conditions of the automation (see e.g., Wickens et al., 2002). However, there can still be instances of boundary conditions and situations where an automated system reaches and exceeds its limits. In such cases, occasional reassessments may occur and further calibration may be needed. Indeed, due to the wide variety of potential situations, individuals must adjust their trust levels whenever they experience something new. This array of diverse and potentially rare scenarios leads to a continuous calibration of trust, representing a dynamic balance and adjustment between trust and automation capabilities. In this context, NDRTs may be seen as a barrier for trust calibration, as they prevent drivers' observations of system behavior.

The foundational theoretical model from Lee and See (2004) links calibration with the Theory of Planned Behavior (TPB; Ajzen, 1991), postulating trust as an attitude and reliance on automation as a behavior. However, this adaptation of trust calibration within the TPB framework overlooks perceived behavioral control, a core dimension of the TPB. Perceived behavioral control refers to an individual's belief that they have control over an action they are performing. Applied to automated driving, we propose an adaptation of this concept which we label perceived behavioral control over automation. We define perceived behavioral control over automation as the expectation that the automated system will operate the vehicle, and that the driver can regain control if required or desired.

To consolidate its integration within trust calibration, we assume that a larger perimeter of perceived behavioral control over automation includes individuals' perceived ability to cope with a situation in case the automation does not operate satisfactorily. Users may or may not be ready to cope with a situation where an automation produces errors, malfunctions or failures. The term “recovered error” denotes the adaptation mechanism that allows road users to cope with complex tasks (Amalberti, 2001), and is used within the road safety literature (Van Elslande, 2003). Some automation errors, malfunctions and failures can be rectified by individuals, such as when they regain control of the automated vehicle following a sensor failure and a takeover request (TOR) is issued. Therefore, error/malfunction/failure recovery should be considered an integral component of trust calibration, positioned within the realm of perceived behavioral control over automation. As a result, perceived behavioral control over automation and error recovery are expected to improve trust calibration. Moreover, we want to stress the importance of experiencing different situations in the process of trust formation/calibration. Deciding whether to rely on an automated system necessitates assessing the automation's capabilities in relation to the current (driving) situation. Consequently, trust may be well-calibrated for certain situations but poorly calibrated for others, particularly those that may occur for the first time. This focus on situation-based trust calibration reveals various potential future research directions that have not been sufficiently addressed so far.

First of all, what characterizes a situation? Referring to Endsley's (1995) definition of situation awareness, we may define a situation as a configuration “of the elements in the environment within a volume of time and space” (p. 65). From a machine perspective, it is relatively easy to identify situations (i.e., the space-time volume is determined by the sensor range and the sensor update frequency). However, there is no agreement about what constitutes a “situation” from an operator's perspective. We assume that different users have a different understanding of questions such as: “When does a situation start/end?”, “What characterizes a unique situation?”, or “When can situations be considered similar?”.

Ultimately, we argue that studies addressing a “calibration of trust” should clearly delineate (1) how trust has been measured in relation to automation performance, (2) if and how trust is conceptually linked to reliance in the given experiment, (3) if and how direct and indirect experience and perceived behavior control were taken into account, and (4) how situations are characterized.

### 2.3 Trust and SAE levels

In discussing the concept of trust within the context of automated vehicles, it is important to recognize the interplay between varying levels of vehicle automation, distinct features, and trust in automation. Mirroring the complex, multi-layered construct of trust in automation, there exists an extensive array of driving automation features, each boasting unique capabilities and limitations. Hence, it is not sufficient to speak about trust in automated vehicles generically. Instead, consideration should be given to the different levels of automation under diverse circumstances.

The vehicle automation levels as described by the Society of Automotive Engineers (SAE, 2021) have become the standard to classify driving automation systems that perform part or all of the dynamic driving task (DDT). It describes six levels of driving automation, ranging from no driving automation (Level 0) to full driving automation (Level 5).

Although the SAE levels are primarily described from an engineering perspective, they also delineate the user's role at each level. A critical distinction exists between Levels 0, 1 and 2, and Levels 3, 4 and 5 in terms of the user's responsibilities. In SAE Levels 0, 1, and 2, the human is always driving and is fully accountable. Even when Level 1 and Level 2 features (jointly known as *driver support features*) are engaged, the human *is* formally always driving, bears complete responsibility, and must constantly supervise these support functions. Conversely, with SAE Levels 3, 4, and 5 features (referred to as *automated driving features*) engaged, the human driver is—sometimes temporarily—*not* driving. A further distinction within SAE Levels 3, 4, and 5 automated driving features is that the human is required to drive when the Level 3 feature requests, whereas Levels 4 and 5 automated driving features do not depend on the human driver to resume control. However, there might still be instances where the driver could be asked to take over.

Generally, examples of such support or automated features include automatic emergency braking, blind spot warning and lane departure warning (SAE Level 0); adaptive cruise control or lane centering (SAE Level 1); a combination of adaptive cruise control and lane centering operating simultaneously (SAE Level 2); a traffic jam chauffeur (SAE Level 3); a local driverless taxi that may or may not have pedals and/or a steering wheel capable of operating in restricted areas (SAE Level 4); and a driverless taxi capable of operating everywhere and in all conditions that a human driver could handle, barring exceptions during extreme weather scenarios (SAE Level 5) (SAE, 2021).

In terms of trust in automation, these distinctions in the driver's responsibilities imply that driver support features and automated driving features must be individually considered when designing for appropriate reliance. For all levels of driving automation, calibrated trust is desirable (Lee and See, 2004). However, the repercussions of miscalibrated trust—overtrust surpassing system capabilities that may result in misuse on one hand, and distrust that falls short of system capabilities possibly leading to disuse on the other hand—differ among features with varying levels of driving automation.

As outlined earlier, SAE Levels 0, 1, and 2 driver support features share the commonality that they must be under the constant supervision of the human driver, who may need to steer, brake, or accelerate to ensure safety (SAE, 2021). This entails that trust calibration is a prerequisite for appropriate reliance and use. Instances of both overtrust/misuse and undertrust/disuse of such systems have been documented (e.g., Malta et al., 2012; Serter et al., 2017; Walker et al., 2018; OVV, 2019).

In contrast to these driver support features, when a SAE Level 3 automated driving feature is engaged, the driver is temporarily relieved of their driving responsibilities, including monitoring the road. However, when a SAE Level 3 automated driving feature indicates it cannot sustain ADS functionality—such as when approaching a work zone or an exit—the human in the driver's seat must resume control within a reasonable timeframe. This requirement distinguishes Level 3 from Level 4 and 5 automated driving features. Consequently, SAE Level 3 features stand out and have sparked particular research interest, even though many argue that Level 3 functionalities will still require a minimal risk maneuver, in the event that a driver is unable to resume control.

Lastly, SAE Level 4 automated Public Transport and taxis and Level 5 automated driving features can be considered together, as the passenger is not required to take control when these features are engaged. The difference between these two levels lies only in the driving conditions they can handle: SAE Level 5 can drive under all conditions in which a trained driver could drive, whereas SAE Level 4 can operate under limited and highly trained conditions and locations. With this in mind, it is less likely for trust in automation to exceed system capabilities and lead to misuse at these levels, making overtrust a less significant concern.

**Figure 2:**
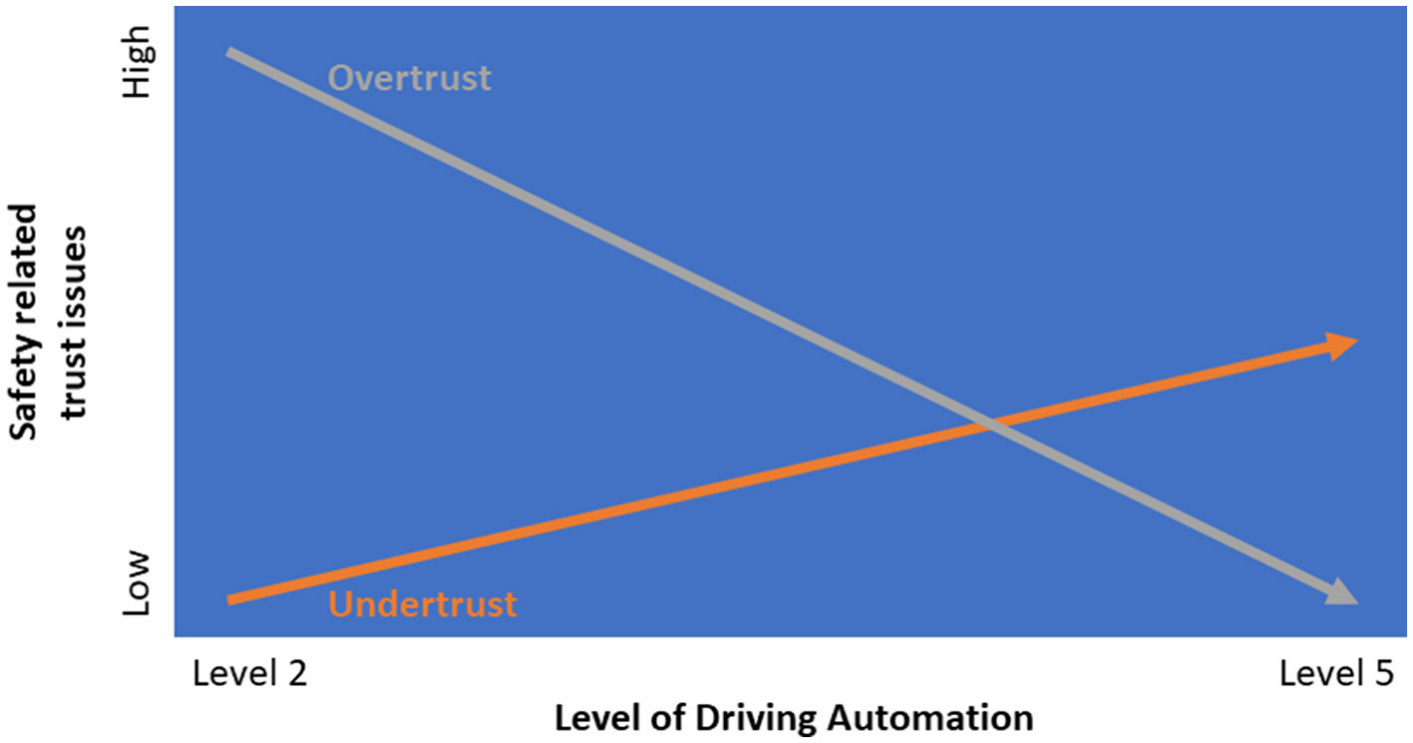
Schematic representation of the relationship between level of driving automation and safety related trust issues. While overtrust (exceeding system capabilities) may lead to misuse, undertrust (falling short of system capabilities) may cause disuse. These two forms of miscalibrated trust (as described by Lee and See, 2004) can hinder successful human-automation collaboration across various levels of driving automation. The figure is not intended to imply that the relationships are linear or direct and should therefore be interpreted with caution.

In summary, we argue that the consequences of (mis)calibrated trust in automation and (in)appropriate reliance are not uniform across all driving automation features, but vary between different SAE Levels. For example, at lower levels of automation overtrust may give rise to serious related safety issues. At higher levels, the main, but less serious risk, may be undertrust and underuse of the system (see Figure 2).

## 3 Assessing trust in automated driving systems

As research on trust in driving automation continues to grow, there has been a corresponding rise in methods, measures and approaches used to operationalize trust. This variety has expanded further due to associated research in other domains such as assisted driving, robotics, and artificial intelligence, leading to a comprehensive toolkit encompassing a wide range of trust measurements. These include self-report (subjective) and direct observation methods, such as measurement scales, single-item ratings, continuous measurement techniques, eye-tracking, behavioral observations, and psychophysiological assessment techniques (for an overview, see Hergeth et al., 2016; Kohn et al., 2021). For example, Hergeth et al. (2016) looked closely into the relationship between self-report (i.e., single-item trust measure) and behavioral (i.e., glance behavior: monitoring frequency) measures, showing a strong relationship between reported trust and resulting reliance behavior in automated driving. However, there were no physiological measures involved in the study. This gap was bridged by the work of Walker et al. (2019b) who added electrodermal activity and related it to glance behavior.

Still, a key concern with many methods used to quantify trust is their lack of objectivity, reliability, and validity. This deficiency can lead to measurements that either roughly approximate trust in automation in a binary and non-multi-layered manner or measure unrelated constructs. Consequently, this may lead to inaccurate conclusions and decisions in designing automated vehicles and associated Human-Machine Interface (HMI) concepts. In the following section, we present criteria and recommendations to aid researchers and practitioners in selecting suitable trust measures—or combinations thereof—for studies on automated driving. The guiding principles in this context are rooted in theoretical considerations and psychometric quality criteria, as outlined by Bühner (2011). From these considerations, we propose a possible framework for assessing various measures of trust in automation.

### 3.1 Theoretical and psychometric considerations for measuring trust in automation

Typical main quality criteria for the construction of psychometric measurement scales (e.g., Trochim, 2001) include (a) objectivity—ensuring consistent scoring across different evaluators and scenarios, (b) reliability—the extent to which a measure is dependable and results in consistent findings, and (c) validity—the soundness of the test's interpretation (does it measure what it is supposed to measure?). These criteria can be subdivided even further for more detailed evaluations (see Table 1). Moreover, when selecting measurements, secondary quality criteria such as standardization, comparability, economy, and usefulness should also be taken into account (Bühner, 2011).

**Table 1:** A framework for assessing frequently used measures of trust in automation.

			**Self-report measures**			**Behavioral measures**		**Psychophysiological measures**		
			**Questionnaires**	**Single-item ratings**	**Continuous measurements with handset controls**	**Eye tracking**	**Interaction behavior**	**EEG**	**Skin conductance**	**fNIRS**
Main quality criteria	Objectivity	Procedure								
		Analysis								
		Interpretation								
	Reliability	Internal consistency								
		Stability								
	Validity	Content validity								
		Construct validity								
		Criterion validity								
Secondary quality criteria	Standardization									
	Comparability									
	Economy									
	Usefulness									

Based on Bühner (2011) and Kohn et al. (2021). For the chosen methods relevant to a specific study setting, cells can be filled in with ratings like “low”, “mid”, or “high”.

All in all, the main criterion for any measurement is construct validity—“the degree to which inferences can legitimately be made from the operationalizations in your study to the theoretical construct” (Trochim, 2001, p. 64). Without construct validity, it is impossible to transfer a measured variable in a study to the underlying theoretical construct. Therefore, the first step toward a high-quality measurement for trust in automation is a sound theoretical understanding of trust processes at a psychological level. This includes a theoretical differentiation of different trust variables (such as trust propensity, trustworthiness, expectation, reliance intention and actual reliance behavior; e.g., Scholz et al., under review) and a clear distinction from related variables (like acceptance or perceived safety and comfort).

Although the terms trust and reliance are frequently used interchangeably, it is crucial to clarify that according to Lee and See's (2004) framework and its extension by Kraus (2020), trust, in combination with other attitudes, can but does not necessarily generate an intention to rely on automation. Whether this results in observable reliance behavior on the automation depends on various contextual factors, such as the workload of the operator or time constraints. In other words, while trust influences reliance, it neither determines it nor can it be considered synonymous with reliance. Therefore, different measures with distinct characteristics need to be employed to assess trust as an attitude (trustworthiness expectation), the intention to rely, and actual reliance behavior.

Furthermore, the propensity to trust in automated technology—viewed as a technology-specific personality trait (e.g., Scholz et al., under review)—requires measurement with a distinct scale. Similarly, trust should not be used as an umbrella term for related but clearly distinct constructs such as technology acceptance (Payre et al., 2021).

Considering the dynamic nature of trustworthiness expectation, several frameworks suggest that both trust and its influence on reliance are part of a dynamic feedback process wherein these variables undergo calibration (see Lee and See, 2004; Hergeth et al., 2016; Kraus, 2020; Walker, 2021). Importantly, trust calibration has been observed even before actual interaction with an automated system (e.g., Hoff and Bashir, 2015; Payre et al., 2017; Kraus et al., 2019; Kraus, 2020).

Taking all this into account, during the interaction with an automated system, information about the outcomes of that interaction is used to update and calibrate expected trustworthiness, which, in turn, significantly influences decision-making in ongoing automation use (Kraus, 2020). In terms of measurement, this calls for repeated trust assessment prior to and during the interaction with automated systems. This allows researchers to understand dynamic trust calibration as an outcome of continuous information updates (Holthausen et al., 2020). Therefore, trust often needs to be measured repeatedly over time and assessed in relation to its developmental trajectory (trust increases and decreases). In this respect, the situational and task-specific nature of trust should be considered (e.g., Lee and See, 2004; Walker et al., 2018; Holthausen et al., 2020; Torggler et al., 2022; Kraus et al., 2023). For example, while an automated vehicle might be generally trusted, the expected trustworthiness of single functions or the functionality of the system in specific critical situations may be diminished.

In this context, the dimensionality of trust is worth discussing. An ongoing debate exists, with some researchers positing mistrust as an additional dimension of trust (e.g., distrust/mistrust; e.g., Lewicki et al., 1998; Harrison McKnight and Chervany, 2001; Spain et al., 2008; Wintersberger et al., 2021), and others claiming that distrust merely represents the lower end of a unidimensional construct (Mayer et al., 1995; Jian et al., 2000; Schoorman et al., 2007; Thielmann and Hilbig, 2015).

Supporting the latter perspective, a second factor often emerges from negatively framed trust items in factorial analyses and could thus be considered a methodological artifact tied to respondent tendencies toward positively vs. negatively framed items. This tendency has been reported in other domains and is noted in basic statistical discussions of psychometric procedures (Wong et al., 2003; Merritt, 2012; Salazar, 2015). Alarcon et al. (2022) provide evidence for this interpretation, showing that the assumption of two dimensions does not withstand an empirical investigation for the propensity to trust.

Another unresolved issue is whether to incorporate dimensions that represent underlying beliefs about trustworthiness or to simply use items that broadly speak to trust. This decision should be made based on the nature of the automated system under investigation and its intended use.

### 3.2 Evaluation of different trust measurements

When choosing methods to measure trust in automation, both primary and secondary quality criteria should be considered and weighed against the specific needs of the situation. For instance, in certain contexts, it is important to achieve the highest possible validity, while in others, economic considerations (the time and effort required to apply a measurement) could be a crucial factor. Table 1 provides an overview of various measures to operationalize trust in automation and outlines criteria for evaluating their advantages and disadvantages. This resource can be used by researchers to make well-informed decisions about the potential methods available. While it includes the most frequently employed methods today, this compilation does not claim to be complete, and it definitely permits the incorporation of further evaluation and comparison techniques.

### 3.3 Recommendations and checklist for increasing the psychometric quality of trust in automation measurements

To enhance the psychometric quality of any selected measurement technique, consider the following recommendations when implementing one or more of the previously mentioned trust measures:

1. Objectivity

1. Standardize measurement procedures by providing clear and comprehensive instructions for both experimenters and participants.
2. Clearly define each stage of data collection, ingest, preparation and analysis, for example, by providing coding schemes, templates, instructions on how to deal with missing data and outliers, and pre-prepared analysis tools.
3. Provide actionable guidance for data interpretation, for example by setting cut-off criteria and supplying comparison data.

2 Reliability

1. If applicable, ensure and verify internal consistency, for instance, by calculating split-half reliability.
2. If possible, assess and control the stability of learned trust, especially in longitudinal studies.

3. Validity

1. Identify the specific trust variable (e.g., dispositional trust, dynamic learned trust) you aim to measure, and select an appropriate measurement method accordingly.
2. Inspect content validity for each research question and before administering the scale.
3. Define the specific trustee or group of trustees (e.g. *all* ADS).
4. Evaluate construct validity.
5. Summarize evaluations to gauge criterion validity.
6. If possible, maintain the original wording of items and answering scales when applying them; do not make changes.

From these theoretical considerations and past research, we can identify several aspects that can serve as best practice guidelines for researchers and practitioners designing experiments. These guidelines, while not comprehensive or entirely distinct, can be useful for identifying typical issues in advance, based on challenges encountered in previous research. Therefore, before selecting a measurement method, consider whether:

- You want to operationalize perceived trustworthiness, reliance intention, reliance behavior, or all of these.
- Your aim is to measure trust at a single point in time (which can only provide a relative evaluation of trust to another system), or to track its formation and calibration over time.
- You want to investigate trust calibration, resolution, temporal specificity or functional specificity.
- Participants should receive prior information about the driving automation you are investigating (Hergeth et al., 2017).
- You should capture an initial level of trust at the beginning of the study (before and / or after first contact; with or without further explanation). However, any inquiry regarding this initial trust level must clearly delineate the type of automated system in question, as well as the specific situations or conditions (such as passenger use, personal vehicle use, public transport, highway driving, or experimental vehicle use, among others).
- Potential timepoints where trust might change can be identified in advance, and plan suitable trust measurement intervals accordingly.
- Psychometric quality matches your study's needs.
- Validated scales can be applied (one-item trust measures should only be used when there is no alternative and findings should be further substantiated in follow-up studies).
- Validated scales should be modified, such as changes to the wording of items or instructions, and if so, whether the validity of the scale used can be adequately accounted for.
- Validated translations of the scale are available.
- It is possible to include in your study behavioral measures of reliance as proxies/indicators of trust.
- You should collect various types of trust measurements, such as questionnaires combined with eye tracking.
- There may be cultural differences among participants, including potential tendencies to provide positive answers to specific questions (e.g., Hergeth et al., 2015).

## 4 Conclusions

Our aim in this paper has been to contribute to a growing body of research on trust in automated vehicles and to provide insights into its conceptualization, measurement, and implications. In line with a vast body of literature, trust needs to be discussed at a level of complexity that agrees with its dynamic and multi-layered nature: it develops and changes over time. Yet, the terminology used to describe this dynamic process is often ambiguous and lacks clarity. We therefore present a concise framework based on previous work (see Figure 1) and provide a use-case example demonstrating its application.

In the framework (see Figure 1), the development of an appropriate level of dynamic learned trust is represented as a cycle in which drivers interact with the automated system in a range of situations, thereby learning how the vehicle behaves and when it can be trusted, and modifying how much and when they can rely on its automated functions. Every time drivers interact with the system, they re-calibrate their trust dynamically and may modify their behavior, based on their updated assessment of the capabilities of the automated system.

Of course, this process involves the accumulation of experience. What counts here, however, is not just the time spent using the system or the distance traveled, but the range of situations in which drivers have been able to assess its behavior and how this experience can be transferred to other situations. In short, it is not just the quantity, but also the quality of drivers' experience that shapes trust in automation. For example, unexpected, rare situations may have a stronger and more lasting impact on trust than situations that are experienced more frequently. It is important, therefore, that studies investigating the calibration of trust in automated vehicles clearly describe how they take account of “experience,” how they characterize the situations to which drivers are exposed, and how trust is measured and conceptually linked to behavior (reliance).

This example points to broader methodological issues. To identify appropriate behavior, it is crucial that researchers evaluate the potential influences of overtrust (or mistrust) and undertrust (or distrust) on system use, taking into account differences in what constitutes inappropriate behavior at different SAE Levels. We observe that methods used to quantify trust often lack objectivity, reliability, and validity. We therefore propose a set of recommendations, aimed at helping researchers to select suitable trust measures.

All this having been said, several key points remain open for future work. First of all, we should clarify the psychological processes through which different trust variables are established and shaped. Clear definitions and variable labels would represent an important step in this direction.

Second, it would be useful to gain a better understanding of the interaction between information and expectations prior to system use, and the impact on initial learned trust. Similarly, we need more research into the role of experience in the development of dynamic learned trust and, in particular, the impact of specific (especially rare) situations.

Third, despite well-established trust in automation scales (e.g., Jian et al., 2000; Chien et al., 2014), we need dynamic measures capable of capturing short-term changes in drivers' dynamic learned trust. Of course, these measures should adhere to the basic principles of objectivity, reliability and validity, highlighted in this paper. Such research could use a combination of self-reports (e.g., rating scales), behavioral observations (e.g., gaze behavior, usage, time on task) and psychophysiological measures (e.g., EEG, EDA, SCR).

Forth, we require more long-term longitudinal studies to shed light on the way trust develops in real-life settings over long periods of time. Most studies on trust in automated vehicles have been conducted in driving simulators, with an average duration of 2 h per participant. Although trust calibration may be observed within a 2-h timeframe, it is likely that calibration in the wild will take days or weeks, depending on the system's frequency of use.

Fifth, we need more studies investigating how well results obtained in driving simulators (where drivers are never at risk of physical harm) transfer to real world situations, in which they face genuine dangers. While engaged in Level 3, individuals hand over their physical integrity to an ADS. Although Walker et al. (2019a) results suggest that even without the risk of physical harm, mid-level driving simulators already elicit a strong sense of presence, results of driving simulator studies should be viewed in the light of a missing crucial aspect for trust development, namely “vulnerability.” Therefore, we need more real-world driving data. This may be collected on test tracks, for the sake of high internal validity, or via field operational tests (FOT) and naturalistic driving studies (NDS), for the sake of increased external validity.

Sixth, we need a better understanding of how experience and trust calibration on one specific system influence expectations about other systems and automated driving in general.

Seventh, identification of the key factors influencing trust is of no practical value unless this knowledge is integrated into interaction concepts for automated vehicles. It is vital that such concepts should foster the emergence of realistic mental models and expectations, facilitating the development of well calibrated trust.

Importantly, our paper does not encompass all potential interventions aimed at enhancing human interactions with automated vehicles. As noted by one of our reviewers, concepts such as swarm intelligence, facilitated by vehicle-to-vehicle (V2V) and vehicle-to-infrastructure (V2I) communication, global controllers overseeing AV operations, interactive feedback provided by the HMI, and emergency bailout buttons can potentially foster user trust. While these interventions fall beyond the scope of our paper, they undeniably present promising avenues for future research.

In conclusion, safe deployment of current (Level 2, Level 3) and future (Levels 4 and 5) commercially available ADS depends on drivers developing appropriate (well-calibrated) levels of trust in the systems. Importantly, even the most experienced drivers are always exposed to a limited range of situations, and will never experience the full range of situations they might encounter in the future. It is essential therefore that they are taught to incorporate the possibility of new and unexpected driving situations in their mental models of system capabilities. We need to develop new methods to achieve this—mainly in driver training and the design of trust-centered interaction concepts. But even if we succeed, improvements will always be possible. Given the potentially lethal consequences of overtrust, research to facilitate such improvements is of vital importance.

## Data availability statement

The original contributions presented in the study are included in the article/supplementary material, further inquiries can be directed to the corresponding author.

## Author contributions

FW: Conceptualization, Funding acquisition, Project administration, Supervision, Writing—original draft, Writing—review & editing. YF: Conceptualization, Writing—review & editing. SH: Conceptualization, Writing—review & editing. JK: Conceptualization, Writing—review & editing. WP: Conceptualization, Writing—review & editing. PW: Conceptualization, Writing—review & editing. MM: Conceptualization, Supervision, Writing—review & editing.
